# Magnetic Resonance Imaging (MRI) of Intratumoral Voxel Heterogeneity as a Potential Response Biomarker: Assessment in a HER2+ Esophageal Adenocarcinoma Xenograft Following Trastuzumab and/or Cisplatin Therapy^1^^2^

**DOI:** 10.1016/j.tranon.2017.03.006

**Published:** 2017-04-26

**Authors:** Connie Yip, Amanda Weeks, Karen Shaw, Musib Siddique, Fuju Chang, David B. Landau, Gary JR. Cook, Vicky Goh

**Affiliations:** ⁎ Department of Cancer Imaging, Division of Imaging Sciences & Biomedical Engineering, King's College London, St Thomas' Hospital, London SE1 7EH, UK; † Department of Radiation Oncology, National Cancer Centre, 11 Hospital Drive 169610, Singapore; ‡ Division of Imaging Sciences & Biomedical Engineering, King's College London, St Thomas' Hospital, London SE1 7EH, UK; § Department of Histopathology, Guy's and St Thomas' NHS Foundation Trust, St Thomas' Hospital, London SE1 7EH, UK; ¶ Department of Clinical Oncology, Guy's and St Thomas' NHS Foundation Trust, St Thomas' Hospital, London SE1 7EH, UK; # Clinical PET Imaging Centre, Guy's and St Thomas' NHS Foundation Trust, St Thomas' Hospital, London SE1 7EH, UK; ⁎⁎ Department of Radiology, Guy's and St Thomas' NHS Foundation Trust, St Thomas' Hospital, London SE1 7EH, UK

## Abstract

We evaluated magnetic resonance imaging (MRI) voxel heterogeneity following trastuzumab and/or cisplatin in a HER2+ esophageal xenograft (OE19) as a potential response biomarker. OE19 xenografts treated with saline (controls), monotherapy, or combined cisplatin and trastuzumab underwent 9.4-T MRI. Tumor MRI parametric maps of T1 relaxation time (pre/post contrast), T2 relaxation time, T2* relaxation rate (R2*), and apparent diffusion coefficient obtained before (TIME0), after 24 hours (TIME1), and after 2 weeks of treatment (TIME2) were analyzed. Voxel histogram and fractal parameters (from the whole tumor, rim and center, and as a ratio of rim‐to‐center) were derived. Tumors were stained for immunohistochemical markers of hypoxia (CA-IX), angiogenesis (CD34), and proliferation (Ki-67). Combination therapy reduced xenograft growth rate (relative change, ∆ +0.58 ± 0.43 versus controls, ∆ +4.1 ± 1.0; *P* = 0.008). More spatially homogeneous voxel distribution between the rim to center was noted after treatment for combination therapy versus controls, respectively, for contrast-enhanced T1 relaxation time (90th percentile: ratio 1.00 versus 0.88, *P* = 0.009), T2 relaxation time (mean: 1.00 versus 0.92, *P* = 0.006; median: 0.98 versus 0.91, *P* = 0.006; 75th percentile: 1.02 versus 0.94, *P* = 0.007), and R2* (10th percentile: 0.99 versus 1.26, *P* = 0.003). We found that combination and trastuzumab monotherapy reduced MRI spatial heterogeneity and growth rate compared to the control or cisplatin groups, the former providing adjunctive tumor response information.

## Introduction

Esophageal cancer is the eighth commonest cancer worldwide [1]. Outcome remains poor with a 5-year overall survival rate of 18% in all patients [2]. Neoadjuvant chemotherapy and chemoradiation have been shown to improve survival in patients with resectable cancer [3], [4], [5]. The addition of trastuzumab, an anti‐human epidermal growth factor receptor 2 (HER2) monoclonal antibody, to standard chemotherapy improves overall survival in HER2-positive advanced gastroesophageal adenocarcinoma compared to chemotherapy alone [6].

One of the challenges in clinical practice is how best to image the spatial and temporal intratumoral changes with treatment. Qualitative (decrease in metabolic activity) and semiquantitative (standardized uptake value: uptake/[injected dose/patient weight]) metabolic assessment with [^18^F] fluorodeoxyglucose positron emission tomography has improved on the sensitivity and specificity of computed tomography size-based response assessment in the neoadjuvant setting [7]. However, with the advent of hybrid positron emission tomography/magnetic resonance imaging (MRI) scanners, there has been renewed interest in the additional potential of MRI for assessing esophageal cancer [8], [9]. MRI reflects the soft tissue anatomy well [10], has no radiation burden, and offers a multiparametric capability beyond anatomical evaluation. For example, diffusion-weighted and dynamic contrast-enhanced MRI sequences reflecting intratumoral water diffusion (a surrogate for cellular volume) and vascularization (a surrogate for angiogenesis), respectively, have shown clinical potential following chemotherapy and/or chemoradiation in esophageal cancer [11], [12], [13], [14].

We hypothesize that conventional chemotherapy (cisplatin) and targeted therapy (trastuzumab) cause distinctive phenotypic and biological changes within the tumor spatially over the course of treatment, reflecting their specific mechanisms of action and downstream effects. This treatment-related change may be captured by image heterogeneity analysis on a per-voxel basis, also known as image texture analysis. We suggest that *in vivo* spatial changes in the image texture may augment standard size-based response evaluation and complement histopathological evaluation in clinical practice [15].

Thus, as proof of principle, we aimed to evaluate the sequential changes in intratumoral MRI spatial heterogeneity following trastuzumab and/or cisplatin therapy in a HER2-expressing esophageal adenocarcinoma xenograft (OE19) and to compare this with histopathological changes in angiogenesis, hypoxia, and cellular proliferation.

## Materials and Methods

### Xenograft Model

All experiments were approved by our institutional review board and performed in accordance with the UK Home Office Animals (Scientific Procedures) Act 1986. The HER2-expressing OE19 cells were cultured in RPMI 1640 medium (Sigma-Aldrich, St Louis, MO) supplemented with 2 mM L-glutamine and 10% fetal bovine serum. Cells were incubated at 37°C in a humidified environment with 5% carbon dioxide. Approximately 5 × 10^6^ OE19 cells, in serum-free media mixed with Cultrex basement membrane extract (Trevigen Inc., Gaithersburg, MD) 1:1 to a final volume of 8 mg/ml, were injected subcutaneously into the right flanks of 6- to 8-week-old female severe combined immunodeficient mice (Charles River Laboratories International, Inc.). Animals were monitored, and bidimensional measurements were obtained using a digital caliper. Once the tumors reached a minimum diameter of 8 mm, the animals were treated with intraperitoneal sterile saline (control group), cisplatin 4 mg/kg body weight once a week (cisplatin monotherapy group), trastuzumab 20 mg/kg twice a week (trastuzumab monotherapy group), or a combination of cisplatin 4 mg/kg once a week and trastuzumab 20 mg/kg twice a week (combination therapy group). Tumors were excised after 2 weeks of therapy and were fixed in 10% buffered formalin before being embedded in paraffin for immunohistochemistry.

### *In Vivo* Imaging

MRI was performed with a 9.4-T MRI system (Bruker, Karlsruhe, Germany). The animals in each of the four groups (controls, cisplatin, trastuzumab, and combination) were anesthetized using inhalational isoflurane (2%-4%) and 1 l/min oxygen during MRI. A high-resolution T1 relaxation time map with and without intravenous administration of gadopentetate dimeglumine 0.1 mmol/kg (Magnevist; Bayer HealthCare Pharmaceuticals Inc., Germany), T2 relaxation time map, R2* map, and apparent diffusion coefficient (ADC) map were generated from the MRI acquisitions. Table 1 shows the MR acquisition parameters. Each animal was imaged at three time points: before treatment (TIME0), 24 hours after the first intraperitoneal therapy injection (TIME1), and after completion of 2 weeks of intraperitoneal treatment (TIME2).

**Table 1:** MRI Acquisition Parameters

Parameters	T1-Weighted	T2-Weighted	Diffusion-Weighted	R2*
Pulse sequence	Rapid acquisition rapid echo with variable repetition time (RARE-VTR)	Multislice multiecho (MSME)	Echo planar	Multigradient echo (MGE)
Respiratory gating	No	No	No	Yes
Repetition time (ms)	193.44, 478.4, 878.44, 1555.502, 5000	2500	3000	1500
Echo time (ms)	6.16	13 TEs from 7.4 to 98.02	16.81	First TE 3.5 ms with 4-ms echo spacing
Field of view (mm)	30 × 30	30 × 30	30 × 30	30 × 30
Matrix	256 × 256	256 × 256	128 × 128	256 × 256
Number of signal averages	1	1	4	1
*b* values (s/m^2^)	Not applicable	Not applicable	100, 250, 500, 750, 1000	Not applicable
Spatial resolution (mm/voxel)	0.117 × 0.117	0.117 × 0.117	0.234 × 0.234	0.117 × 0.117
Slice thickness (mm)	1	1	1	1

### Image Analysis

All the MR parametric maps were analyzed using in-house software implemented under the MATLAB (The MathWorks Inc., Natick, MA) platform. Whole tumor volumes of interest (VOIs) were delineated by a single observer (C. Y.). Tumor volumes were derived from the T1 images. Three-dimensional differential analysis of the tumor rim and tumor center was also performed given the inhomogeneous tumor morphology. The tumor rim was defined as the outer 3.5 mm (T1, T2, R2* maps) or outer 4.6 mm (ADC map) of a tumor, reflecting the slightly different voxel size of the ADC map. For tumors with volumes below the median value, a 2.3-mm rim was required to allow adequate sampling of the rim versus center on the ADC maps.

First-order statistical histogram and fractal analysis of T1, T2, R2*, and ADC voxels were analyzed, and the following parameters were derived for whole tumor, tumor rim, and tumor center on each parametric map, respectively: mean, median, maximum, range, standard deviation, kurtosis, skewness, entropy, energy, 10th percentile, 25th percentile, 75th percentile, 90th percentile, mean fractal dimension, and fractal lacunarity (Table A1 in Appendix 1) [16], [17], [18].

The ratio of all MR parameters (apart from skewness) between tumor rim and tumor center at each time point was defined as:

$$\frac{\text{Absolute value in tumor}\;\mathrm{rim}\;\mathrm{at}\;\text{TIMEn}}{\text{Absolute value}\;\mathrm{at}\;\text{tumor center}\;\mathrm{at}\;\text{TIMEn}}$$

where *n* = 0, 1, or 2.

Ratios equal to 1 indicate homogeneous voxel distribution, whereas ratios greater or less than 1 indicate more inhomogeneous voxel distribution between rim and center.

As skewness could be positive or negative, the absolute differences between the skewness values within tumor rim and tumor center were obtained instead of the above ratios:

$$\left(\text{Absolute}\;\text{value}\;\text{in}\;\text{tumor}\;\mathrm{rim}\;\mathrm{at}\;\text{TIMEn}\right)-\left(\text{absolute}\;\text{value}\;\text{in}\;\text{tumor}\;\text{center}\;\mathrm{at}\;\text{TIMEn}\right)$$

where *n* = 0, 1 or 2.

Ratio = 0 indicates homogeneous voxel distribution, whereas ratios greater or less than 0 indicate more inhomogeneous voxel distribution between rim and center.

Relative proportional volumetric changes (∆) between TIME2-TIME0 were calculated as follow:

$$\frac{\text{Absolute value}\;\mathrm{at}\;\text{TIME}2-\text{absolute value}\;\mathrm{at}\;\text{TIME}0}{\text{Absolute value}\;\mathrm{at}\;\text{TIME}0}$$

### Pathological Tumor Response

Excised tumors were fixed in 10% buffered formalin before being embedded in paraffin for immunohistochemistry. Contiguous 5-μm sections of formalin-fixed paraffin-embedded tumors were obtained for hematoxylin and eosin (H&E) and immunohistochemical staining of CA-IX, CD34, and Ki-67 (see Appendix 2 for further details). The ratios of necrotic and fibrotic areas over the whole tumor H&E section were quantified and categorized as minimal (ratio < 0.1), moderate (ratio 0.1-0.5) and extensive (ratio > 0.5). The tumor regression grade (TRG) was assessed by a pathologist (F. C.) as follows: TRG 1, absence of residual cancer and extensive fibrosis; TRG2, presence of occasional cancer cells scattered through the fibrosis; TRG3, increased residual cancer cells but fibrosis still predominated; TRG4, residual cancer cells outgrowing fibrosis; and TRG5, absence of regressive changes [19].

### Histological Quantification

The CA-IX hypoxia fraction (HF), Ki-67 proliferative fraction (PF), and CD34 microvessel density (MVD) were quantified (see Appendix 2 for further details).

The ratios of positive immunohistochemical staining between tumor rim and center were defined as:

$$\frac{\mathrm{HF},\mathrm{PF},\text{or}\;\mathrm{MVD}\;\text{in tumor}\;\mathrm{rim}\;}{\mathrm{HF},\mathrm{PF},\text{or}\;\mathrm{MVD}\;\text{in tumor center}}$$

### Statistical Analysis

The Kruskal-Wallis test was used to compare the MRI and histopathological whole tumor parameters and ratios between the rim and center between the treatment groups. A Bonferroni correction factor of 5 was applied to account for multiple MR sequences used. A less conservative correction factor was used due to the exploratory nature of this study. A *P* value < 0.01 was considered statistically significant. All statistical analysis was performed using the IBM SPSS Statistics software version 22 (IBM, Armonk, NY).

## Results

From a total of 27 animals, 4 (1 from the cisplatin, 3 from the combination groups) did not have TIME2 imaging due to logistical reasons but were included in the TIME1-TIME0 MR analysis. The remaining 23 animals were included in all analyses (5 in the control, 5 in the cisplatin, 5 in the trastuzumab, and 8 in the combination groups).

### Tumor Control

**Figure 1:**
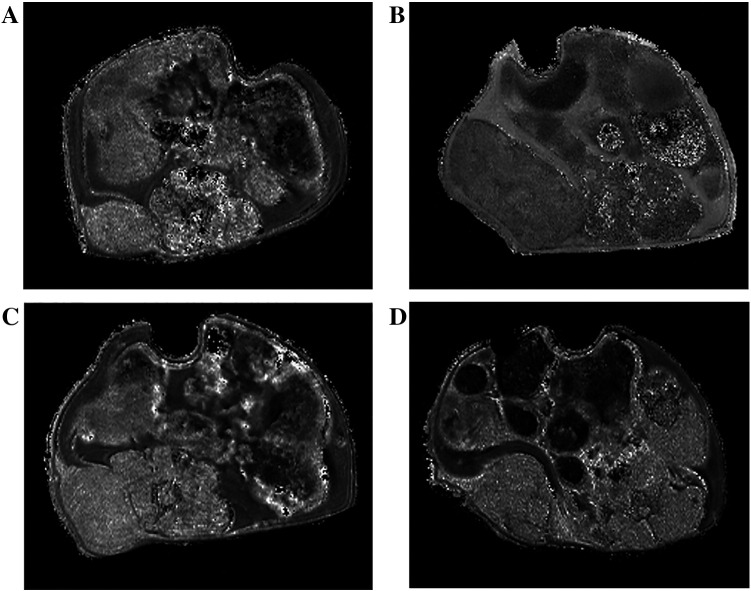
T1 relaxation time maps showing the volumetric changes in control-treated tumor at (A) TIME0 and (B) TIME2, and combination-treated tumor at (C) TIME0 and (D) TIME2.

At TIME2, there was a significantly reduced growth rate in animals treated with trastuzumab (average relative proportional change in volume, ∆: +1.3 ± 1.2) and combination (∆ +0.6 ± 0.4) therapy compared to the control (∆ +4.1 ± 1.0) and cisplatin (∆ +2.4 ± 1.7) groups (*P* = 0.008) (Figure 1).

### Pathological Tumor Response

**Figure 2:**
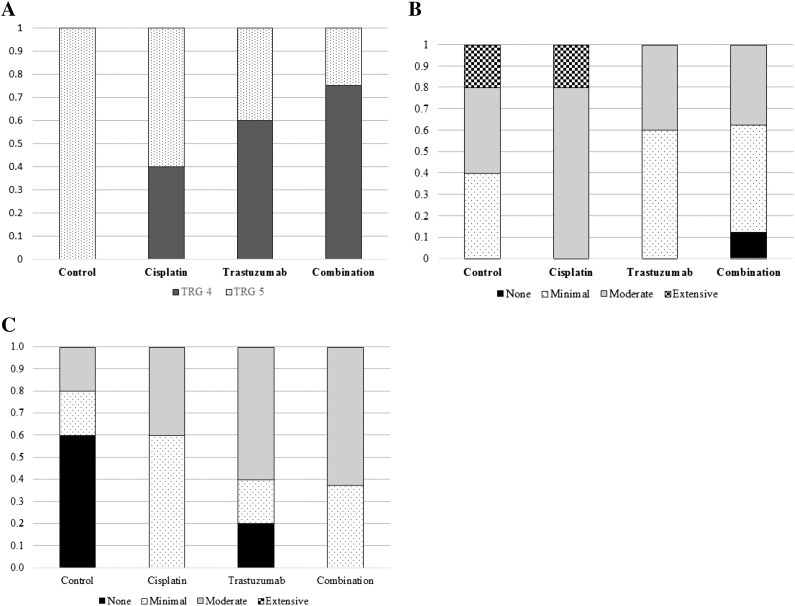
Pathological response according to treatment groups: (A) tumor regression grade, (B) intratumoral necrosis, and (C) intratumoral fibrosis.

**Figure 3:**
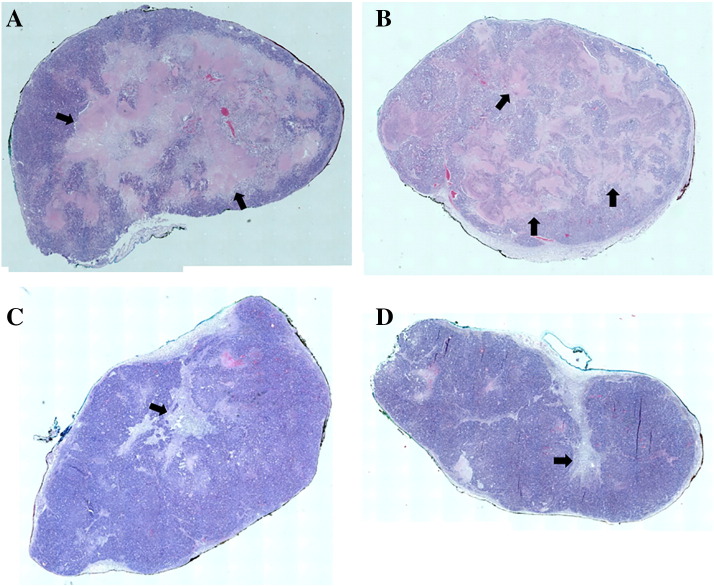
H&E sections showing extensive intratumoral necrosis (black arrows) in (A) cisplatin- and (B) control-treated tumors, and (C, D) evidence of fibrosis (black arrows) in combination-treated tumors.

A greater proportion of tumors in the combination (75%) and trastuzumab (60%) groups showed some degree of pathological response (TRG4) compared to none in the control group (Figure 2*A*). Moderate to extensive intratumoral necrosis was found in all cisplatin-treated tumors (*n* = 5) and the majority of controls (*n* = 3) (Figure 2*B*). In contrast, there was less necrosis in the combination-treated tumors, but this group demonstrated a greater degree of intratumoral fibrosis compared to control and cisplatin groups (Figures 2*C* and 3).

### Intratumoral Spatial Heterogeneity Between Treatment Groups

#### MRI

**Figure 4:**
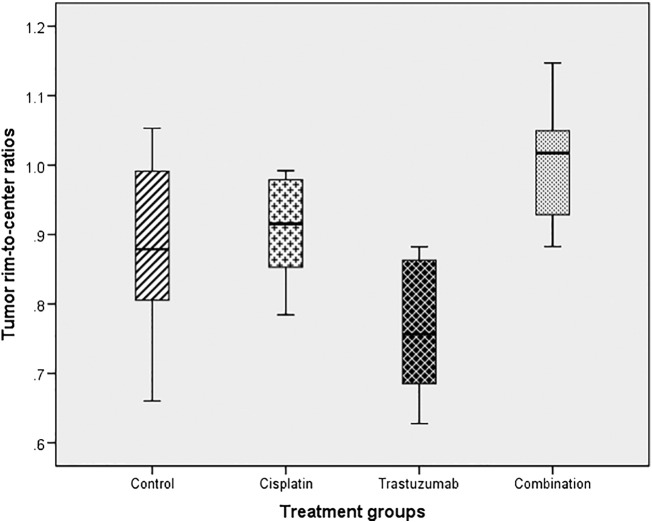
Box plot showing the contrast-enhanced T1 90th percentile tumor rim-to-center ratios at TIME1 between treatment groups.

At TIME1, the distribution of contrast-enhanced T1 90th percentile values was different between the rim and center between treatment groups, with the combination group showing a more homogeneous distribution, whereas T1 values were lower in the rim compared to tumor center in other groups (average ratio 1.00, 95% confidence interval [CI] 0.69-1.07) compared to other groups (average ratio: cisplatin 0.91, 95% CI 0.82-0.99; trastuzumab 0.76, 95% CI 0.63-0.90; control 0.88, 95% CI 0.94-1.06) (*P* = 0.009) (Figure 4). There were no early (TIME1) spatial differences in R2*, T2, and ADC distribution between treatment groups. See Appendix 3 for the results of all MRI parameters.

**Figure 5:**
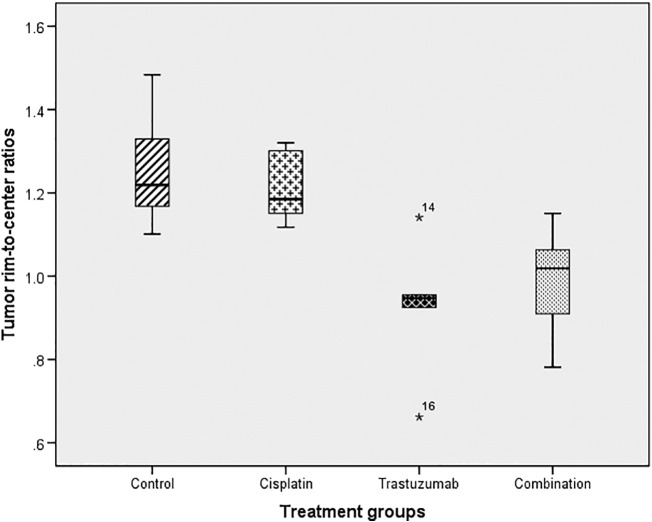
Box plot showing the R2* 10th percentile tumor rim-to-center ratios at TIME2 between treatment groups.

At TIME 2, after completion of treatment, there was a difference in the spatial variation of R2* 10th percentile between the rim and center: the combination (average ratio 0.99, 95% CI 0.89-1.09) and trastuzumab-treated tumors (average ratio 0.93, 95% CI 0.71-1.14) showed more homogeneous R2* values compared to control (average ratio 1.26, 95% CI 1.07-1.45) and cisplatin-treated tumors (average ratio 1.22, 95% CI 1.10-1.33) (*P* = 0.003), where higher rim R2* values were noted (Figure 5).

**Figure 6:**
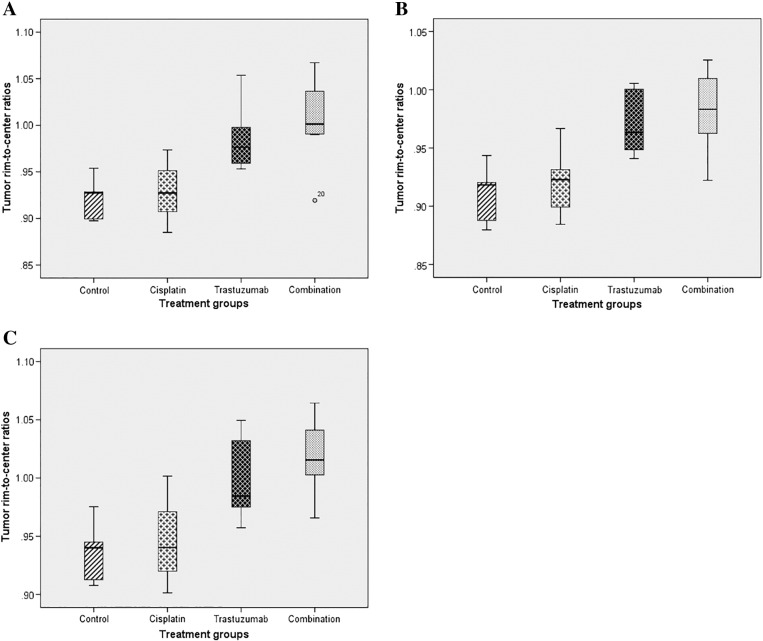
Box plots showing the T2 tumor rim-to-center ratios at TIME2 between treatment groups: (A) mean, (B) median, and (C) 75th percentile.

There were also significant spatial differences in T2 values after treatment. Overall, the combination and trastuzumab groups showed a more homogeneous distribution of T2 values between tumor center and rim compared to the control and cisplatin groups, which showed lower T2 values in the rim compared to the center: T2 mean (average ratio: control 0.92, 95% CI 0.89-0.95; cisplatin 0.93, 95% CI 0.89-0.97; trastuzumab 0.99, 95% CI 0.94-1.04; combination 1.00, 95% CI 0.97-1.04) (*P* = 0.006) (Figure 6*A*), T2 median (average ratio: control 0.91, 95% CI 0.88-0.94; cisplatin 0.92, 95% CI 0.88-0.96; trastuzumab 0.97, 95% CI 0.93-1.01; combination 0.98, 95% CI 0.95-1.01) (*P* = 0.006) (Figure 6*B*), and T2 75th percentile (average ratio: control 0.94, 95% CI 0.90-0.97; cisplatin 0.95, 95% CI 0.90-1.00; trastuzumab 1.00, 95% CI 0.95-1.05; combination 1.02, 95% CI 0.99-1.05) (*P* = 0.007) (Figure 6*C*). There were no spatial ADC differences between treatment groups.

#### Histopathology

**Figure 7:**
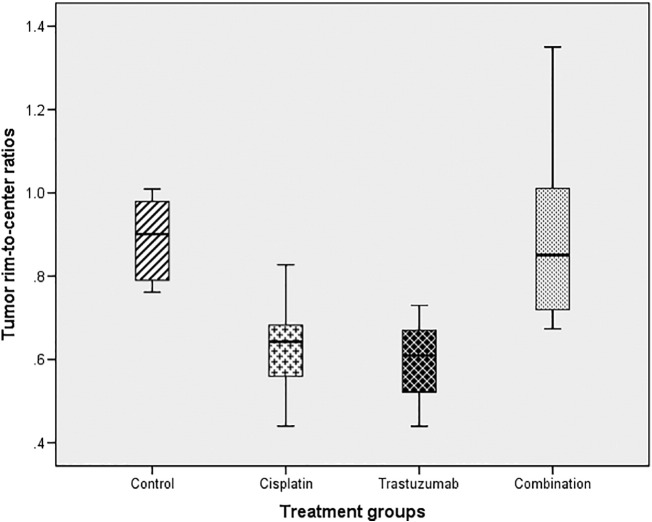
Box plot showing the Ki-67 tumor rim-to-center ratios between the groups.

There was significant spatial difference in Ki-67 (*P* = 0.005) expression between treatment groups (Figure 7). The combination-treated tumors showed a more homogeneous intratumoral Ki-67 expression (average ratio: 0.90, 95% CI 0.71-1.09), suggesting less proliferative spatial variation after treatment compared to the monotherapy groups (cisplatin: 0.63, 95% CI 0.45-0.81 and trastuzumab: 0.59, 95% CI 0.45-0.74). There was no significant difference in spatial heterogeneity of CD34 and CA-IX expression between treatment groups (Table A7 in Appendix 4).

### Whole Tumor Parameters Between Treatment Groups

#### MRI

There were no significant differences in early TIME1 MRI parameters between treatment groups. Posttreatment whole tumor TIME2 T2 SD_H_ was greater in the combination (average 12.16, 95% CI 10.22-14.10) and trastuzumab-treated tumors (average 12.25, 95% CI 7.84-16.65) compared to the cisplatin (average 8.42, 95% CI 7.25-9.61) and control groups (average 9.60, 95% CI 9.00-10.19) (*P* = 0.004). There were no significant differences in whole tumor T1, ADC, and R2* parameters between treatment groups.

#### Histopathology

Whole tumor pathological expression of Ki-67, CD34, and CA-IX did not differ between treatment groups (Table A8 in Appendix 4).

## Discussion

To our knowledge, there has been no published literature evaluating the sequential changes in image spatial heterogeneity (so-called “image texture”) and its association with histopathology in trastuzumab- and/or cisplatin-treated esophageal cancer, both of which form the backbone of systemic therapy in this disease. In our xenograft study, we found spatial differences between the tumor rim and center that were not captured by whole tumor metrics.

A decrease in MRI intratumoral spatial heterogeneity following combination and trastuzumab therapy was observed. In particular, contrast-enhanced T1 relaxation time, T2 relaxation time, and T2* relaxation rate voxels were more uniformly distributed between the tumor rim and center after 2 weeks of intraperitoneal combination and trastuzumab monotherapy. In contrast, untreated controls demonstrated greater voxel heterogeneity with higher R2* values but decreased contrast-enhanced T1 and T2 relaxation times within the tumor rim compared to tumor center, compatible with greater contrast delivery/leakage but increased hypoxia in the rim. Ki-67 expression was also more homogeneous in the combination group. In contradistinction, there was no difference in whole tumor MRI or pathological parameters between the four groups.

While differential tumor rim to center expression of growth factors and response to systemic therapy have been shown in other cancers [15], [20], [21], few studies have investigated imaging with pathology. Eichhorn et al. assessed the imaging and pathological tumor microcirculation in tumor rim and center after vascular disruptive agent (VDA) therapy (ZD6126) in the Lewis lung carcinoma xenograft model [21]. The group performed contrast-enhanced ultrasound at 24 hours after VDA therapy and stained the excised tumors for CD31. They showed that tumor rim had greater rate of signal increase, change in signal intensity from baseline to initial peak, and also CD31 MVD after treatment compared to the tumor center.

In a study that evaluated epidermal growth factor receptor (EGFR) expression in 386 resected colorectal cancers, 74% of tumors showed concordance of EGFR expression between tumor center and rim [20]. A greater proportion of tumor rim was EGFR positive (58%) compared to 46% of tumor centers. In addition, a greater EGFR expression in tumor rim relative to tumor center, which was found in 25% of patients, was associated with inferior survival in this patient cohort.

Nguyen et al. evaluated the pathological spatial variation of vascular, angiogenic, hypoxia, and epithelial-to-mesenchymal transition marker expressions between tumor rim and center in a murine colorectal metastatic model with and without VDA therapy (OXi4503) [15]. In the untreated tumor model, the tumor center had greater CD34 microvessel density compared to the tumor rim. However, vessels in the rim were more mature and stable as shown by increased alpha smooth muscle actin staining of pericytes compared to the tumor center. The rim was also less hypoxic as defined by pimonidazole staining and showed greater mesenchymal transition compared to the tumor center. Following treatment with OXi4503, the tumor rim showed less vascular endothelial cell and tumor cell apoptosis, and higher cellular proliferation compared to the tumor center up to 24 hours posttreatment. These changes disappeared as tumors regained proliferative capacity after 5 days. These results suggest that differential inherent and treatment-related imaging and/or pathological spatial changes may have prognostic and predictive implications. An increase in peripheral epithelial-to-mesenchymal transition could lead to increased invasive and metastatic potential. Similarly, residual remnant rim of proliferative tumor cells may harbor resistant subclones resulting in treatment failure.

Overall, our results appear consistent with previous studies that showed differential spatial treatment-related effects associated with targeted therapy. In our study, untreated tumors demonstrated more heterogeneous MR voxels between the tumor rim and center with higher R2* relaxation rate (associated in part with increased deoxyhemoglobin concentration) but reduced postcontrast T1 relaxation time (related to greater vascularization/vascular permeability) and T2 relaxation time values at the periphery, whereas combination-treated tumors were more homogeneous.

Imaging heterogeneity assessed by agnostic approaches has a clinical potential to identify responders/nonresponders at an earlier time point for intensification of treatment in esophageal cancer. Nevertheless, we acknowledge that it will be challenging to assess differential rim and center imaging characteristics in an early primary esophageal tumor. However, a significant proportion of patients will present with locally advanced tumors. We also acknowledge that our study has several limitations. A relatively small sample size will limit the statistical power for some observations. The imaging time points used in this study were chosen to represent early and late response assessment, similar to that used in the clinical setting [22], [23]. The lack of significant MRI changes at 24 hours post–first therapy in this study suggests that there were no “hyperacute” MRI changes that could be detected following intraperitoneal trastuzumab and cisplatin therapy in a mouse xenograft model. It is possible that this could be due to the route of drug delivery used. Hence, we suggest that an early imaging time point in a xenograft model treated with intraperitoneal treatment protocol such as the one used in this study may be best performed after a week of therapy to elicit clinically relevant early imaging treatment changes to parallel what is done in the clinical setting. Lastly, our study was performed on a 9.4-T small-animal MR scanner and not a clinical scanner, but our study should be regarded as proof of principle of the potential differential therapeutic MRI effects.

In conclusion, our study showed that the combination of trastuzumab and cisplatin therapy was most effective in slowing tumor growth rate in a HER2+ esophageal adenocarcinoma xenograft. Our findings suggest that trastuzumab therapy, either alone or in combination with cisplatin, resulted in greater MR spatial homogeneity compared to untreated/cisplatin-treated tumors. Assessment of intratumoral spatial heterogeneity across the rim and center has the potential to augment standard imaging assessment following neoadjuvant chemotherapy in esophageal cancer.

The following are the supplementary data related to this article.

Appendix 1 Summary description and relevant formulae for first order histogram statistics and fractal features. Appendix 1

Appendix 2 Methods and materials (supplementary data). Appendix 2

Appendix 3 The statistical significance of the difference in the MRI tumor rim-to-center ratios between treatment groups at TIME1 and TIME2. Appendix 3

Appendix 4 Methods and materials (supplementary data). Appendix 4
