# Supplementary material for: Magnetic Resonance Imaging (MRI) of Intratumoral Voxel Heterogeneity as a Potential Response Biomarker: Assessment in a HER2+ Esophageal Adenocarcinoma Xenograft Following Trastuzumab and/or Cisplatin Therapy
Source: Transl Oncol. 2017 Apr 26;10(3):459–67. doi: 10.1016/j.tranon.2017.03.006 (PMC5408154; doi:10.1016/j.tranon.2017.03.006)
Supplement: Appendix 1 — Summary description and relevant formulae for first order histogram statistics and fractal features. [file mmc1.docx]

**APPENDIX 1**

**Table A1: Summary description and relevant formulae for first order histogram statistics and fractal features**

| **Parameter** | **Description** | **Formula** |
| --- | --- | --- |
| **Mean** | Describes the average of all voxel signal intensities. | $\frac{1}{n}\sum_{(x,y)\in R} a(x,y)$  Where *n* = the total number of voxels in *R*, the region-of-interest within the image *a(x,y).* |
| **Median** | Describes the signal intensity value in the middle when voxels are ranked in ascending order. |  |
| **Skewness** | Measures signal asymmetry & deviation from a normal distribution.  Skewness>0: right skewed, values concentrated on the left of the mean.  Skewness<0: left skewed, values concentrated on the right of the mean.  Skewness=0: normal distribution | $\frac{n}{(n-1)(n-2)}\frac{\sum_{(x,y)\in R} {a\left( x,y \right)-\bar{a}]}^{3}}{{[sd\left( a \right)]}^{3}}$  Where *n* = the total number of voxels in the region of interest *R* within the image *a(x,y)*; *sd* = standard deviation; *ā* is the mean value within *R*. |
| **Kurtosis** | Describes the "peak" of a distribution.  Kurtosis>3: sharper peak than a normal distribution.  Kurtosis<3: flatter peak than a normal distribution.  Kurtosis=3: normal distribution. | $\frac{n}{(n-1)(n-2)(n-3)}\frac{\sum_{(x,y)\in R} [a(x,y)-\overline{a}]^{4}}{[sd(a)]^{4}}-3\frac{(n-1)^{2}}{(n-2)(n-3)}$  Where *n* = the total number of voxels in the region-on-interest, *R* within the image *a(x,y)*; *sd* = standard deviation; *ā* is the mean value within *R*. |
| **Energy** | Measures voxel signal distribution. High energy is noted in homogeneous voxels. | $\sum_{i=1}^{imax} {[p\left( i \right)]}^{2}$  Where *i* is the voxel value (between i=1 to *i_max_* in the region of interest and *p(i)* the probability of the occurrence of that voxel value. |
| **Entropy** | Measures voxel randomness. Low entropy is noted in homogeneous voxels. | $\sum_{i=1}^{imax} [p(i)ln[p(i)]$  Where *i* is the voxel value (between *i* =1 to *i_max_* in the region of interest and *p(i)* the probability of the occurrence of that voxel value. |
| **Mean fractal dimension** | Measures the texture of a fractal, a self similar pattern. A higher fractal dimension corresponds to greater roughness. | $\bar{D}=\frac{\sum_{i=1}^{N} D_{i}}{N}$  Where *N* is the number of slices and $D_{i}$ is the fractal dimension for the $i^{th}$ slice. |
| **Lacunarity** | Measures the amount of “gaps” in the image/object. If a fractal has large “gaps”, it has high lacunarity. | $\frac{\left[ {\sum_{i=1}^{N} D_{i}^{2}}/N \right]}{\left[ \left( \frac{\sum_{i=1}^{N} D_{i}}{N} \right)^{2} \right]}-1$  Where *N* is the number of slices and $D_{i}$ is the fractal dimension for the $i^{th}$ slice. |
