# Supplementary material for: Magnetic Resonance Imaging (MRI) of Intratumoral Voxel Heterogeneity as a Potential Response Biomarker: Assessment in a HER2+ Esophageal Adenocarcinoma Xenograft Following Trastuzumab and/or Cisplatin Therapy
Source: Transl Oncol. 2017 Apr 26;10(3):459–67. doi: 10.1016/j.tranon.2017.03.006 (PMC5408154; doi:10.1016/j.tranon.2017.03.006)
Supplement: Appendix 2 — Methods and materials (supplementary data). [file mmc2.docx]

**APPENDIX 2**

**Methods & materials (supplementary data)**

**Immunohistochemistry**

Contiguous 5μm sections of formalin-fixed paraffin-embedded tumors were obtained. Following deparaffinisation and rehydration, endogenous peroxidase activity was blocked using 3% hydrogen peroxide in PBS for 10 minutes. Heat induced antigen retrieval was performed using citrate acid buffer (10mM Citric Acid, 0.05% Tween 20, pH 6.0) for all stains apart from GLUT1. Non-specific binding was blocked using 10% normal goat serum (Dako, Germany) diluted in PBST for 20 minutes at room temperature for CA-IX and Ki-67 whereas 10% normal rabbit serum (Dako, Germany) was used for CD34. Following that, the sections were incubated with the following primary antibodies at room temperature for one hour: rabbit polyclonal anti-CAIX (1:25 dilution; Millipore, Temecula, USA), rabbit polyclonal anti-Ki67 (1:300 dilution; Abcam, Cambridge, UK) and rat monoclonal anti-CD34 (1:5 dilution; Hycult Biotech, Uden, Netherlands). Sections were then exposed to biotinylated polyclonal goat anti-rabbit secondary antibody (1:200 dilution; Dako, Germany) for CA-IX and Ki-67, or biotinylated anti-rat secondary antibody for CD34 (1:200 dilution; Vector Laboratories Inc, Burlingame, USA) at room temperature for 30 minutes. Sections were subsequently incubated with the Avidin-Biotin Complex (Vectastain^®^, ABC Reagent, Burlingame, USA) at room temperature for 30 minute followed by DAB (3, 3’-diaminobenzidine) peroxidase substrate (Dako, Germany) for stain development. The sections were then counterstained with hematoxylin, dehydrated and mounted for microscopy.

**Immunohistochemistry quantification**

Tumor sections were scanned and tiled at 100x magnification using a Leica LEITZ DMRB microscope linked to a camera and motorized stage which was operated using the Objective Imaging’s Surveyor 8.1 software (Objective Imaging Ltd, Cambridge, UK). Histological sections were analyzed using ImageJ 1.49v software (National Institutes of Health, UK). Positive immunohistochemistry staining was quantified using colour thresholding by adjusting the hue, saturation and brightness to identify the positive brown stain. CA-IX hypoxic fraction (HF) was defined as below:

$$\frac{\text{Total area of CA-IX labelled tumor }}{\text{Total area of whole tumor section }}$$

The proliferative fraction (PF) was defined as the ratio of the total tumor area stained with Ki-67 over the total area of whole tumor section excluding any necrotic component:

$$\frac{\text{Total area of Ki-67 labelled tumor }}{\text{Total area of whole }\text{tumor}\text{ section excluding necrotic component}}$$

Microvessel density (MVD) as identified by CD34 staining was used to quantify angiogenesis. A 0.1 x 0.1cm grid was superimposed on the tumor section and any blood vessels and/or endothelial cells that were positively stained with CD34 were counted in each 0.01cm^2^ box. MVD was defined as the average number of blood vessels and/or endothelial cells per 0.01cm^2^ at 100x magnification:

$$\frac{\text{Number of blood vessels and/or endothelial cells}}{\text{Number of }\text{0.01cm}^{\text{2}}\text{ boxes in whole tumor section}}$$
