# Supplementary material for: Magnetic Resonance Imaging (MRI) of Intratumoral Voxel Heterogeneity as a Potential Response Biomarker: Assessment in a HER2+ Esophageal Adenocarcinoma Xenograft Following Trastuzumab and/or Cisplatin Therapy
Source: Transl Oncol. 2017 Apr 26;10(3):459–67. doi: 10.1016/j.tranon.2017.03.006 (PMC5408154; doi:10.1016/j.tranon.2017.03.006)
Supplement: Appendix 3 — The statistical significance of the difference in the MRI tumor rim-to-center ratios between treatment groups at TIME1 and TIME2. [file mmc3.docx]

**APPENDIX 3**

**The statistical significance of the difference in the MRI tumor rim-to-center ratios between treatment groups at TIME1 and TIME2**

**Table A2: Non-contrast enhanced T1 relaxation time**

| **MRI parameters** | ***p*-values** | |
| --- | --- | --- |
|  | **TIME1** | **TIME2** |
| Mean | 0.524 | 0.679 |
| Median | 0.409 | 0.665 |
| Maximum | 0.841 | 0.722 |
| Range | 0.818 | 0.730 |
| Standard deviation | 0.635 | 0.993 |
| Skewness | 0.842 | 0.743 |
| Kurtosis | 0.719 | 0.605 |
| Entropy | 0.580 | 0.637 |
| Energy | 0.621 | 0.587 |
| 10^th^ percentile | 0.786 | 0.453 |
| 25^th^ percentile | 0.581 | 0.728 |
| 75^th^ percentile | 0.456 | 0.755 |
| 90^th^ percentile | 0.431 | 0.894 |
| Mean fractal dimension | 0.559 | 0.215 |
| Fractal lacunarity | 0.082 | 0.151 |

**Table A3: Contrast-enhanced T1 relaxation time**

| **MRI parameters** | ***p*-values** | |
| --- | --- | --- |
|  | **TIME1** | **TIME2** |
| Mean | 0.025 | 0.029 |
| Median | 0.291 | 0.098 |
| Maximum | 0.028 | 0.240 |
| Range | 0.038 | 0.296 |
| Standard deviation | 0.014 | 0.071 |
| Skewness | 0.877 | 0.919 |
| Kurtosis | 0.831 | 0.744 |
| Entropy | 0.558 | 0.617 |
| Energy | 0.406 | 0.824 |
| 10^th^ percentile | 0.139 | 0.102 |
| 25^th^ percentile | 0.226 | 0.127 |
| 75^th^ percentile | 0.027 | 0.022 |
| 90^th^ percentile | **0.009** | 0.030 |
| Mean fractal dimension | 0.212 | 0.086 |
| Fractal lacunarity | 0.582 | 0.483 |

**Table A4: T2 relaxation time**

| **MRI parameters** | ***p*-values** | |
| --- | --- | --- |
|  | **TIME1** | **TIME2** |
| Mean | 0.742 | **0.006** |
| Median | 0.209 | **0.006** |
| Maximum | 0.847 | 0.402 |
| Range | 0.821 | 0.432 |
| Standard deviation | 0.686 | 0.036 |
| Skewness | 0.692 | 0.163 |
| Kurtosis | 0.707 | 0.214 |
| Entropy | 0.601 | 0.342 |
| Energy | 0.630 | 0.465 |
| 10^th^ percentile | 0.792 | 0.082 |
| 25^th^ percentile | 0.548 | 0.030 |
| 75^th^ percentile | 0.314 | **0.007** |
| 90^th^ percentile | 0.506 | 0.010 |
| Mean fractal dimension | 0.284 | 0.143 |
| Fractal lacunarity | 0.257 | 0.527 |

**Table A5: R2***

| **MRI parameters** | ***p*-values** | |
| --- | --- | --- |
|  | **TIME1** | **TIME2** |
| Mean | 0.811 | 0.014 |
| Median | 0.512 | 0.034 |
| Maximum | 0.146 | 0.145 |
| Range | 0.180 | 0.103 |
| Standard deviation | 0.472 | 0.186 |
| Skewness | 0.627 | 0.330 |
| Kurtosis | 0.606 | 0.357 |
| Entropy | 0.478 | 0.486 |
| Energy | 0.282 | 0.579 |
| 10^th^ percentile | 0.527 | **0.003** |
| 25^th^ percentile | 0.690 | 0.010 |
| 75^th^ percentile | 0.810 | 0.042 |
| 90^th^ percentile | 0.749 | 0.061 |
| Mean fractal dimension | 0.204 | 0.164 |
| Fractal lacunarity | 0.256 | 0.354 |

**Table A6: ADC**

| **MRI parameters** | ***p*-values** | |
| --- | --- | --- |
|  | **TIME1** | **TIME2** |
| Mean | 0.232 | 0.124 |
| Median | 0.726 | 0.186 |
| Maximum | 0.582 | 0.748 |
| Range | 0.789 | 0.576 |
| Standard deviation | 0.766 | 0.415 |
| Skewness | 0.675 | 0.170 |
| Kurtosis | 0.575 | 0.156 |
| Entropy | 0.996 | 0.404 |
| Energy | 0.869 | 0.500 |
| 10^th^ percentile | 0.063 | 0.058 |
| 25^th^ percentile | 0.196 | 0.166 |
| 75^th^ percentile | 0.373 | 0.114 |
| 90^th^ percentile | 0.702 | 0.111 |
| Mean fractal dimension | 0.198 | 0.198 |
| Fractal lacunarity | 0.388 | 0.388 |
