# Supplementary material for: Magnetic Resonance Imaging (MRI) of Intratumoral Voxel Heterogeneity as a Potential Response Biomarker: Assessment in a HER2+ Esophageal Adenocarcinoma Xenograft Following Trastuzumab and/or Cisplatin Therapy
Source: Transl Oncol. 2017 Apr 26;10(3):459–67. doi: 10.1016/j.tranon.2017.03.006 (PMC5408154; doi:10.1016/j.tranon.2017.03.006)
Supplement: Appendix 4 — Methods and materials (supplementary data). [file mmc4.docx]

**APPENDIX 4**

**Table A7: Difference in tumor rim-to-center ratios of hypoxia, angiogenesis and proliferation immunohistochemistry**

| **IHC markers** | **Tumor rim-to-center ratios (average)** | | | | ***p*-values** |
| --- | --- | --- | --- | --- | --- |
|  | **Combination** | **Trastuzumab** | **Cisplatin** | **Control** |  |
| **CA-IX** | 1.32 | 0.87 | 2.06 | 2.16 | 0.023 |
| **Ki-67** | 0.90 | 0.59 | 0.63 | 0.94 | **0.005** |
| **CD34** | 0.70 | 0.64 | 1.16 | 1.20 | 0.255 |

**Table A8: Difference in whole tumor immunohistochemistry markers of hypoxia, angiogenesis and cellular proliferation.**

| **IHC markers** | **Combination** | **Trastuzumab** | **Cisplatin** | **Control** | ***p*-values** |
| --- | --- | --- | --- | --- | --- |
| **CA-IX HF** | 0.013 | 0.008 | 0.004 | 0.004 | 0.030 |
| **Ki-67 PF** | 0.149 | 0.138 | 0.156 | 0.270 | 0.051 |
| **CD34 MVD** | 37.3 | 23.5 | 18.1 | 18.1 | 0.014 |
